# Data on dimer formation between importin α subtypes

**DOI:** 10.1016/j.dib.2016.03.080

**Published:** 2016-04-01

**Authors:** Yoichi Miyamoto, Masahiro Oka

**Affiliations:** Laboratory of Nuclear Transport Dynamics, National Institutes of Biomedical Innovation, Health and Nutrition, 7-6-8 Saito-Asagi, Ibaraki, Osaka 567-0085, Japan

**Keywords:** Nuclear transport, Importin α, Nuclear localization signal, Dimer

## Abstract

This article describes data related to the research article titled “Functional characterization of importin α8 as a classical nuclear localization signal receptor” [1]. A GST pull-down assay showed that both importin α1 and α8, which are classical nuclear localization signal (cNLS) receptors, can form a dimer with importin α6, α7, or α8. Importin α8 has higher dimer-forming ability than importin α1. In addition, our data show that either importin α1 or importin α8 can form a heterodimer with importin α3, which exists in a preformed complex with cNLS substrates such as the conventional SV40TNLS or the p53 protein, resulting in the release of the cNLS substrates from importin α3.

**Specifications Table**

**Table:** 

Subject area	Biology
More specific subject area	Nucleocytoplasmic transport
Type of data	Figure
How data was acquired	GST pull-down, western blot
Data format	Raw image
Experimental factors	Bacterially expressed and purified recombinant proteins
Experimental features	Bound proteins precipitated by GST pull-down assay were subjected to SDS-PAGE and detected by immunoblotting
Data source location	Osaka, Japan
Data accessibility	Data are accessible in this article only

**Value of the data**

• These data are valuable to researchers interested in the molecular mechanisms by which the importin α-cNLS substrate complex dissociates in the nucleus.
• These data show that importin α1 and α8 have substantial differences in dimer-forming ability, despite both proteins belonging to the same subfamily.
• These data provide a new insight into the function of nuclear-localized importin αs.

## 1 Data

**Fig. 1:**
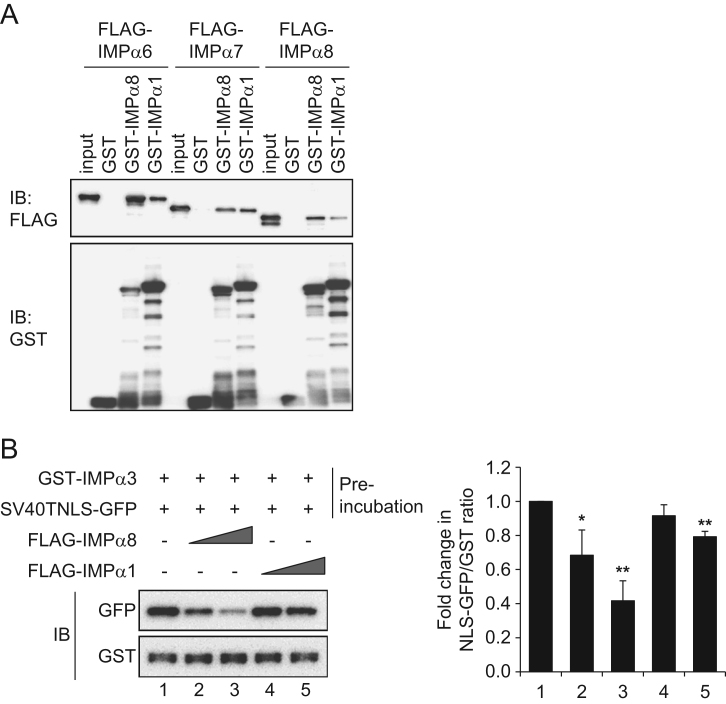
Western blot analysis indicates that importin α subtypes have the potential to form heterodimers with other importin αs. (A) GST-importin α8 (IMPα8) and GST-importin α1 (IMPα1) were incubated with FLAG-importin α6 (IMPα6), FLAG-importin α7 (IMPα7), or FLAG-importin α8 (IMPα8) recombinant proteins. Bound proteins were detected by anti-FLAG antibody or anti-GST antibody, respectively. FLAG-tagged importin αs (0.625 pmol) were loaded as an input. (B) GST-importin α3 (IMPα3) immobilized on GSH beads was preincubated with SV40TNLS-GFP (Preincubation), and then an equal or 10 times higher amount of FLAG-importin α8 or FLAG-importin α1 was added. Left panels: representative immunoblot (IB) images of the NLS-GFP and GST-importin α3 bands. Right panels: relative fold changes in the NLS-GFP/GST ratio in the presence of either importin α8 or α1, which were normalized to the control condition (without FLAG-importin αs). The results are from three independent experiments and have been presented as mean ± SEM. The numbers 1–5 correspond to the lane numbers in the left panels. **p<0.01, *p<0.05; Student׳s *t*-test.

**Fig. 2:**
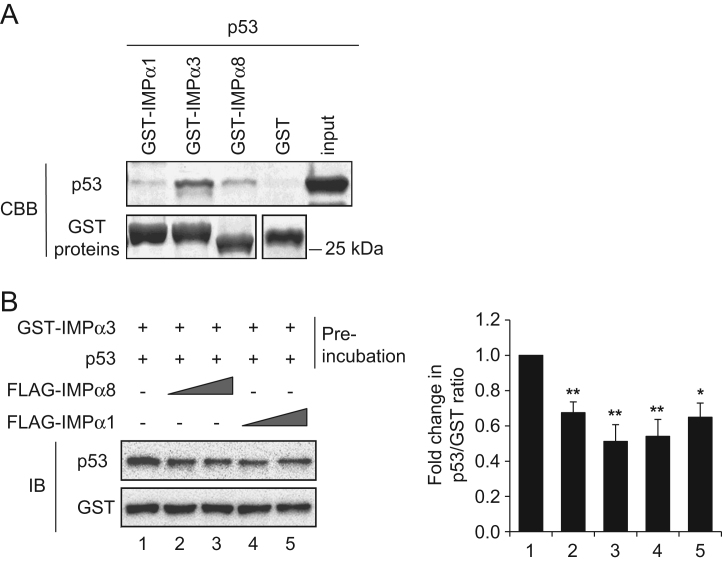
Either importin α8 or α1 dissociates the importin α3-p53 complex by dimer formation with importin α3. (A) p53 preferentially binds to importin α3 rather than importin α1 or α8. Pull-down assays were performed with the p53 recombinant protein and with GST-importin α1 (IMPα1), GST-importin α3 (IMPα3), or GST-importin α8 (IMPα8) immobilized on GSH beads. After incubation at 4 °C for 1 h, the beads were washed and bound proteins were analyzed by SDS-PAGE. Proteins are detected by Coomassie staining. p53 (10 pmol) was loaded as an input control. (B) GST-importin α3 (IMPα3) immobilized on GSH beads was preincubated with a five times higher amount of p53 to form a complex (Preincubation). Then, increasing amounts of FLAG-importin α8 or FLAG-importin α1 (equal or 10 times higher amount than GST-IMPα3) were added. Bound proteins were analyzed by western blotting using the antibodies indicated. Left panels: representative immunoblot images of the p53 and GST-importin α3 bands. Right panels: relative fold changes in the p53/GST ratio in the presence of either FLAG-importin α8 or FLAG-importin α1, which were normalized to the control condition (without FLAG-importin αs). The results are from three independent experiments and have been presented as mean ± SEM. The numbers 1–5 correspond to the lane numbers described in the left panels. **p<0.01, *p<0.05; Student׳s *t*-test.

To examine whether importin αs can directly bind with other importin α subtypes, FLAG-tagged importin α6, α7, or α8 recombinant proteins were incubated with either GST-importin α8, or GST-importin α1, and analyzed by western blotting (Fig. 1A). To investigate the effect of heterodimerization of importin αs on its substrate binding, either an importin α3-SV40TNLS complex, or an importin α3-p53 complex was incubated with increasing amounts of FLAG-importin α8, or α1 recombinant proteins, and analyzed by western blotting (Figs. 1B and 2).

## 2 Experimental design, materials and methods

### 2.1 Plasmid construction

The N-terminus FLAG-tagged cDNAs encoding full-length human importin α6 (KPNA5, NM_012316) or full-length human-importin α7 (KPNA6, NM_002269) were amplified from either HEK293 cells or MRK-nu-1 cells (JCRB Cell Bank, Osaka, Japan) by PCR using the following primers: importin α6 Forward: 5′-CCCGAATTCCGCCATGGACTACAAGGACGACGACGACAAGATGGATGCCATGGCTAGTCC-3′ and importin α6 Reverse: 5′-CCCGCGGCCGCCTCGAGTTAAAGTTGAAATCCATCC-3′ or importin α7 Forward: 5′-CCCGAATTCCGCCATGGACTACAAGGACGACGACGACAAGATGGAGACCATGGCGAGC-3′ and importin α7 Reverse: 5′-CCCGCGGCCGCCTCGAGTTATAGCTGGAAGCCCTCC-3′. The PCR program was as follows: 2 min at 94 °C followed by 40 cycles of 30 s at 98 °C and 15 min at 68 °C. The PCR products were digested with *Eco*RI and *Not*I, and then subcloned into the pGEX6P3 plasmid (GE Healthcare, Tokyo, Japan). The construct integrity of pGEX6P3/FLAG-h-importin α6 and pGEX6P3/FLAG-h-importin α7 was confirmed by DNA sequencing using the following primers: pGEX 5′ sequencing primer: 5′-GGGCTGGCAAGCCACGTTTGGTG-3′, pGEX 3′ sequencing primer: 5′-CCGGGAGCTGCATGTGTCAGAGG-3′, importin α6 sequencing primer: 5′-GCATCTGGAACTTTTCTGCATACC-3′, or importin α7 sequencing primer: 5′-GTACATTACAGTTTGAAGCTGCCT-3′.

The human-importin α8 (KPNA7, NM_001145715) cDNA with the FLAG-tag at the N-terminus was amplified by PCR from the pcDNA5/3xFLAG-h-importin α8 plasmid [1]. The primers were as follows: importin α8 Forward: 5′-CCCGAATTCCGCCATGGACTACAAGGACGACGACGACAAGATGCCGACCTTAGATGCTCC-3′ and importin α8 Reverse: 5′-CCCGCGGCCGCCTCGAGCTATTTTTTTGCTAAGC-3′. The PCR program was the same as that described above. The PCR products were inserted into *Eco*RI and *Not*I sites of pGEX6P3, and then sequenced using either the pGEX 5′ or pGEX 3′ sequencing primer and the importin α8 sequencing primer: 5′-CAACATCGCTTCAGGGACTTCG-3′.

The construct encoding SV40 large T antigen NLS (PPKKKRKVED, pGEX6P2-SV40TNLS-GFP) was subcloned from pGEX2T-SV40TNLS-GFP [2]. The plasmids pGEX6P3/FLAG-human-importin α1 (KPNA2), pGEX6P2-mouse-importin α2 (KPNA2, which we referred to as m-importin α1), and pGEX2T-human-importin α3 (KPNA4, Qip1) were obtained as described previously [1], [3], [4].

The human cDNA encoding the tumor protein p53 (NM_000546) was amplified from MCF7 cells by PCR performed using the following primers: p53 Forward: 5′-CACGGATCCATGGAGGAGCCGCAGTCAGATC-3′ and p53 Reverse: 5′-GGACTCGAGTCAGTCTGAGTCAGGCCCTTCTG-3′. The PCR program was as follows: one cycle of 2 min at 94 °C; 40 cycles of 15 s at 94 °C, 30 sec at 64 °C, and 1 min 20 s at 68 °C; and one cycle of 10 min at 68 °C. The PCR product was subcloned into the *Bam*HI and *Xho*I sites of pGEX6P1, and then verified by sequencing.

### 2.2 Recombinant protein purification

Recombinant proteins fused to GST were purified as follows: The expression vectors were transformed into *Escherichia coli* Rosetta, and then the cells were grown at 37 °C in LB medium containing 50 μg/mL ampicillin. Expression was induced by addition of 1 mM isopropyl-β-d-thiogalactopyranoside (IPTG), followed by incubation at 20 °C for 12 h. The bacteria were lysed in lysis buffer (50 mM Tris–HCl at pH 8.3, 500 mM NaCl, 1 mM EDTA, 2 mM dithiothreitol (DTT), 0.2 mM phenylmethylsulfonyl fluoride (PMSF), 1 μg/mL aprotinin (Nacalai Tesque, Kyoto, Japan), 1 μg/mL pepstatin (Peptide Institute, Osaka, Japan), and 1 μg/mL leupeptin (Peptide Institute)) by freeze–thawing twice and passing through a French press (model: FA-078, SLM Instruments, Rochester, NY, USA). The cell lysates were sonicated using a Sonifier 250 (Branson, Danbury, CT, USA), and centrifuged at 20,400*g* at 4 °C for 30 min. The resultant supernatant was incubated with glutathione-Sepharose 4B beads (GSH beads, GE Healthcare, Tokyo, Japan) at 4 °C for 12 h. After the GSH beads were washed five times with lysis buffer, GST-tagged proteins were eluted with elution buffer (20 mM glutathione, 100 mM Tris–HCl at pH 8.3, 100 mM NaCl, 1 mM EDTA, 2 mM DTT, and 1 μg/mL each of aprotinin, pepstatin, and leupeptin). Cleavage of GST from the GST-fused protein was performed using PreScission protease (GE Healthcare, Piscataway, NJ, USA) with 10 units/mg of fusion protein at 4 °C for 12 h in cleavage buffer (50 mM Tri-HCl at pH 7.0, 150 mM NaCl, 1 mM EDTA, 1 mM DTT, and 1 µg/mL each of aprotinin, leupeptin, and pepstatin). Finally, the purified proteins were dialyzed against dialysis buffer (20 mM HEPES at pH 7.3, 110 mM CH_3_COOK, 2 mM DTT, and 1 μg/mL each of aprotinin, pepstatin, and leupeptin) and concentrated by ultrafiltration using Amicon Ultra centrifugal filter units (Merck Millipore, Tullagreen, Ireland).

### 2.3 GST pull-down assay

Fig. 1A: Bacterially produced FLAG-h-importin α6, α7, and α8 recombinant proteins (100 pmol each) were incubated with GST, GST-h-importin α8 (KPNA7), or GST-m-importin α1 (KPNA2) immobilized on GSH beads in 200 μL of transport buffer (TB; 20 mM HEPES at pH 7.3, 110 mM potassium acetate, 2 mM magnesium acetate, 1 mM EGTA, 1 mM DTT, 500 μM PMSF, and 1 μg/mL each of aprotinin, pepstatin, and leupeptin) with 0.1% Triton X-100 at 4 °C for 1 h. After washing five times with TB containing 0.1% Triton X-100, the beads were suspended in sodium dodecyl sulfate–polyacrylamide gel electrophoresis (SDS-PAGE) loading buffer (50 mM Tris–HCl at pH 6.8, 34.7 mM SDS, 50% glycerol, 25% β-mercaptoethanol, and bromophenol blue). Bound proteins were analyzed by western blotting with specific antibodies described.
Fig. 1B: GST-h-importin α3 (50 pmol) immobilized on GSH beads was incubated with the SV40TNLS substrate (SV40TNLS-GFP, 50 pmol) at 4 °C for 1 h. After washing the beads to remove unbound proteins, either 50 pmol or 500 pmol of FLAG-h-importin α8 or FLAG-h-importin α1 was mixed with the importin α3-SV40TNLS complex at 4 °C for 1 h. The beads were then washed five times with TB containing 0.1% Triton X-100 and suspended in SDS-PAGE loading buffer. Bound proteins were analyzed by western blotting with specific antibodies described.
Fig. 2A: GST-h-importin α1, α3, or α8 (50 pmol each) immobilized on GSH beads were incubated with the p53 protein (50 pmol) in 200 μL TB containing 0.1% Triton X-100 at 4 °C for 1 h. After washing the beads to remove unbound proteins, bound proteins were subjected to 10% SDS-PAGE and stained with Coomassie Brilliant Blue (CBB).
Fig. 2B: GST-h-importin α3 (50 pmol) immobilized on GSH beads was incubated with the p53 protein (250 pmol) in 200 μL TB containing 0.1% Triton X-100 at 4 °C for 1 h. After washing the beads, either 50 pmol or 500 pmol of FLAG-h-importin α8 or FLAG-h-importin α1 was added to the importin α3-p53 complex at 4 °C for 1 h. The beads were then washed five times with TB containing 0.1% Triton X-100, and bound proteins were analyzed by western blotting with the specific antibodies described below.

### 2.4 Antibodies

The following antibodies were used for western blotting: anti-FLAG M2 antibody (F1804, 0.1 μg/mL, Sigma-Aldrich, St. Louis, MO, USA), anti-GST antibody (sc-138, 0.04 μg/mL, Santa Cruz Biotechnology, Texas, USA), anti-GFP antibody (M048-3, 0.1 μg/mL, MBL, Nagoya, Japan), anti-p53 (FL-393) antibody (sc-6243, 0.04 μg/mL, Santa Cruz Biotechnology), and horseradish peroxidase (HRP)-conjugated anti-mouse or anti-rabbit IgG secondary antibodies (1: 10,000 dilution, Jackson ImmunoResearch Lab., West Grove, PA, USA)

### 2.5 Western blotting

Samples were loaded on a 10% SDS-PAGE gel, and the separated proteins in the gel were transferred onto an Immobilon-P membrane (PVDF membrane; Merck Millipore, Darmstadt, Germany) using a semi-dry transfer blotting system (Trans-Blot Turbo Transfer System, BioRad Laboratories, Inc., Hercules, CA, USA). The membrane was blocked with blocking buffer consisting of 3% skim milk in Tris-buffered saline (TBS; TAKARA BIO, Shiga, Japan) with 0.05% Tween (TBS-T) for 1 h. The membrane was probed with primary antibodies diluted in Can Get Signal Immunoreaction Enhancer Solution 1 (TOYOBO, Osaka, Japan) at 4 °C overnight, and then incubated with the HRP-conjugated secondary antibody diluted in Can Get Signal Immunoreaction Enhancer Solution 2 (TOYOBO) at room temperature (RT) for 1 h. After the membrane was washed with TBS-T, it was developed with Chemi-Lumi One L or Super (Nacalai Tesque). The intensity of each western blot signal was quantified by Image J software (http://rsbweb.nih.gov/ij/).
