# Supplementary material for: Data on dimer formation between importin α subtypes
Source: Data Brief. 2016 Apr 1;7:1248–53. doi: 10.1016/j.dib.2016.03.080 (PMC4865633; doi:10.1016/j.dib.2016.03.080)
Supplement: Supplementary file 1 — Supplementary material [file mmc1.doc]

**Conflict of interest**

The authors declare that there is no conflict of interest.
